# The impact of antibiotic exposure on obesity and metabolic phenotypes via the gut microbiota

**DOI:** 10.3389/fmicb.2026.1782016

**Published:** 2026-04-14

**Authors:** Baogang Zhu, Ce Fu, Dawei Fan, Yan Wang, Pengfang Sheng, Jianghua Feng, Xiaopeng Wang

**Affiliations:** 1 The First Clinical Medical College, Gansu University of Chinese Medicine, Lanzhou, China; 2 Department of General Surgery, Gansu Provincial Hospital, Lanzhou, China

**Keywords:** antibiotics, gut microbiota, metabolomics, obesity, probiotics

## Abstract

Antibiotics are among the most commonly used anti-infective agents in modern medicine. However, their long-term effects on the gut microbiome have attracted increasing attention. Epidemiological studies and animal experiments in recent years suggest that antibiotic exposure can disrupt the structure and function of the gut microbiota, thereby affecting host energy metabolism, fat deposition, and immune homeostasis. Such disruptions may contribute to the development of obesity and related metabolic phenotypes. Different classes of antibiotics exert markedly distinct effects on the gut microbiota. Broad-spectrum antibiotics such as macrolides, lincosamides, and fluoroquinolones often induce more pronounced and prolonged microbial alterations, whereas the effects of certain β-lactam antibiotics appear relatively transient. Antibiotic-induced gut dysbiosis can influence host metabolism through multiple mechanisms, including reduced short-chain fatty acid production, disrupted bile acid metabolism, impaired intestinal barrier function, and chronic low-grade inflammation. These alterations may promote fat accumulation, insulin resistance, and disruption of immune homeostasis. Early-life antibiotic exposure occurs during a critical developmental window for gut microbiota maturation and may exert more profound effects on long-term metabolic health. Recent advances in multi-omics technologies have further illuminated the complex interaction network among antibiotics, the microbiome, and host metabolism. Microecological intervention strategies, such as probiotics and synbiotics, show potential for improving metabolic abnormalities associated with antibiotic-induced dysbiosis. However, their efficacy is strain-specific, and the overall effect size remains limited. This review summarizes current research progress on how antibiotic exposure influences obesity and metabolic phenotypes through the gut microbiota, outlines the underlying mechanisms, and discusses potential applications of microbiological intervention strategies. It also provides insights into antibiotic-related metabolic risks and future precision intervention approaches.

## 1 Introduction

Obesity has become a major global public health challenge and is closely associated with metabolic diseases such as type 2 diabetes, cardiovascular disease, and non-alcoholic fatty liver disease (Lingvay et al., 2024; Assumpção et al., 2022). As a key regulator of host metabolism, the gut microbiota is recognized as playing a significant role in the onset and progression of obesity. Previous studies have indicated that alterations in the gut microbiota can influence energy metabolism, fat accumulation, and immune function, which are core mechanisms underlying obesity and its complications (Hu et al., 2024; Crovesy et al., 2020; Sasidharan Pillai et al., 2024).

Antibiotics, as commonly used clinical medications, play a vital role in the treatment of infections. However, their widespread use is also recognized as an external factor contributing to gut microbiota dysbiosis (Anthony et al., 2022). Antibiotic administration is closely associated with alterations in gut microbial composition and function, which may in turn lead to metabolic disorders (Meng et al., 2021). Particularly during early life, the impact of antibiotic exposure on gut microbiota development and long-term metabolic health has attracted considerable attention (Vliex et al., 2024). Although probiotic interventions show potential in alleviating dysbiosis, their efficacy is influenced by host genotype, baseline microbiota status, and the type of antibiotic used (Lukasik et al., 2022). Therefore, elucidating the mechanisms by which antibiotic exposure affects obesity and metabolic health through the gut microbiota is crucial for guiding rational clinical prescribing and optimizing intervention strategies for metabolic disorders.

## 2 The association between antibiotic-mediated gut dysbiosis and obesity

### 2.1 Link between antibiotic exposure and obesity

Recent epidemiological and experimental studies suggest a potential association between antibiotic exposure and the development of obesity, particularly during early life (Schell and Carmody, 2025; Cox and Blaser, 2015). Existing evidence indicates that this association depends not only on antibiotic use itself, but also on several factors, including the exposure window, exposure intensity, and antibiotic spectrum (Shan et al., 2022; Rasmussen et al., 2018; Miller et al., 2018).

A multicenter case-control study found that children exposed to antibiotics during school age exhibited a significantly increased risk of overweight or obesity, particularly when exposed to antibiotics such as florfenicol originating from environmental or food-chain sources (Li et al., 2022). Similarly, a study on environmental antibiotic exposure revealed that elevated urinary levels of tetracycline and sulfamethoxazole were associated with an increased risk of childhood obesity, with this association being more pronounced at low to moderate exposure levels (Çelebi et al., 2024). These findings suggest that low-dose, chronic antibiotic exposure from the food chain or the environment may have potential metabolic implications. Beyond environmental exposure, antibiotic exposure during pregnancy and early life has also been associated with an increased risk of childhood obesity. A retrospective cohort study using large population-based data demonstrated a dose-response relationship between the number of antibiotic classes used, the cumulative number of exposure episodes, earlier age at first exposure, and the occurrence of childhood obesity (Leong et al., 2020). Meanwhile, some studies suggest that the effects of different antibiotic classes are not uniform (Table 1). Broad-spectrum antibiotics, such as fluoroquinolones and macrolides, exhibit relatively stronger associations with obesity risk (Vallianou et al., 2021; Kenyon et al., 2020; Chagas et al., 2023). However, after further adjustment for familial genetic and environmental confounders, this association was attenuated in some analyses (Wilkins and Reimer, 2021), suggesting that antibiotics may act more as amplifying factors in obesity development rather than as independent causative agents.

**Table 1:** Summary of microbiome and metabolic effects associated with different classes of antibiotics.

Antibiotic class	Representative drugs	Target mechanism	Antimicrobial spectrum	Core microbiota alterations	Metabolic effects	Representative studies
β-lactams	Penicillin, amoxicillin, cephalosporins	Inhibition of bacterial cell wall synthesis	Gram-positive and some Gram-negative bacteria	Perturbation of bacterial and bacteriophage communities; reduction of early colonizing taxa such as *Lactobacillus* under early-life low-dose exposure	Altered metabolic profiles; early-life low-dose exposure associated with increased adiposity and altered intestinal lipid metabolism signaling	Cox et al., 2014; Shelton et al., 2023; d'Humières et al., 2024
Tetracyclines	Chlortetracycline, doxycycline	Inhibition of protein synthesis (30S ribosomal subunit)	Broad-spectrum	Restructuring of gut microbial community and altered SCFA-related metabolism; no consistent taxonomic pattern reported	Subtherapeutic exposure associated with increased fat mass and altered energy harvest	Cho et al., 2012; Schell and Carmody, 2025; Vallianou et al., 2021
Macrolides	Azithromycin, tylosin	Inhibition of protein synthesis (50S ribosomal subunit)	Broad-spectrum	Long-lasting alterations in microbial community structure; some compositional changes may persist for several years	Associated with increased obesity risk; experimental models show lipid accumulation and reduced adipose thermogenesis	Mulder et al., 2020; Yu et al., 2022; Ni et al., 2025
Fluoroquinolones	Ciprofloxacin	Inhibition of DNA gyrase and topoisomerase IV	Strong activity against gram-negative bacteria	Rapid reduction in microbial diversity and restructuring of gut microbial communities; accompanied by resistome alterations	Associated with altered growth and obesity risk in some epidemiological studies; microbiome-mediated metabolic mechanisms remain incompletely understood	Anthony et al., 2022; Shan et al., 2022; Yaffe et al., 2025

Animal studies provide compelling evidence for the association between antibiotics and obesity-related metabolic disorders. In mouse models, subtherapeutic (low-dose) antibiotic administration has been consistently shown to increase fat mass. Cho et al. (2012) confirmed that early exposure to low-dose penicillin, vancomycin, or chlortetracycline led to increased fat mass and altered short-chain fatty acids (SCFAs) production without significantly reducing total microbial density. Cox et al. (2014) further showed that low-dose penicillin exposure during pregnancy or weaning induced increased fat mass, impaired immune development, and persistent metabolic alterations. Notably, transplantation of microbiota from antibiotic-treated donors into germ-free recipients partially reproduced the fat accumulation phenotype, confirming a microbiome-mediated causal mechanism. Subsequent studies have further elucidated potential pathways linking antibiotic-induced dysbiosis to metabolic dysfunction. Shelton et al. (2023) found that low-dose penicillin exposure reduced Lactobacillus abundance, thereby decreasing phenylpyruvate production in the small intestine. This, in turn, modulated the PPAR-γ signaling pathway under high-fat diet conditions, thereby promoting lipid accumulation. These findings suggest that moderate disruption of the gut microbiota during critical developmental periods may shift host energy balance toward fat storage. Conversely, high-dose antibiotics typically produce different metabolic outcomes by drastically reducing microbial density. In adult mice, extensive microbiota depletion under certain conditions can mitigate obesity-related phenotypes or improve metabolic parameters, highlighting the dose- and time-dependent nature of antibiotic effects. Liu et al. (2021) investigated how prolonged high-fat diets diminished the efficacy of bactericidal antibiotics, confirming that diet-induced microbiome alterations reduced antibiotic bactericidal activity *in vivo*. Their findings revealed that after microbial depletion, the therapeutic disparity between standard-diet and high-fat-diet mice disappeared, substantiating the existence of a microbiome-dependent mechanism.

In summary, evidence from animal models indicates that the metabolic consequences of antibiotic exposure depend on the dosage, timing of exposure, and developmental stage of the host. During early life, moderate disruption of the gut microbiota may promote fat deposition through microbiome-mediated metabolic reprogramming, whereas in adulthood, severe microbial depletion may produce distinctly different or even opposite metabolic effects.

However, it is important to note that some studies have not accounted for multiple confounding factors that may influence the observed outcomes. For example, maternal body mass index (BMI), dietary habits, socioeconomic status, and infection severity may simultaneously affect both the likelihood of antibiotic use and the incidence of obesity. Additionally, reverse causality should be considered, as obese individuals may experience increased antibiotic use due to a heightened risk of infection. Future studies should rigorously control for these confounding factors to accurately assess the true association between antibiotic exposure and obesity.

### 2.2 Effects of antibiotic exposure on gut microbiota

Antibiotics represent one of the major exogenous factors that disrupt the human gut microbiome, with their effects manifesting across three levels: alterations in community structure, functional changes, and recovery processes (Pennycook and Scanlan, 2021). Previous studies have indicated that following antibiotic exposure, the gut microbiota typically exhibits rapid declines in α-diversity and alterations in β-diversity (Fishbein et al., 2023; Anthony et al., 2022). However, post-treatment recovery does not necessarily equate to a return to the original steady state. Substitution among bacterial strains, persistent abnormalities in certain metabolic functions, and changes in the reservoir of antibiotic resistance genes may all leave long-term “ecological imprints” within the gut (Fishbein et al., 2023).

**Figure 1:**
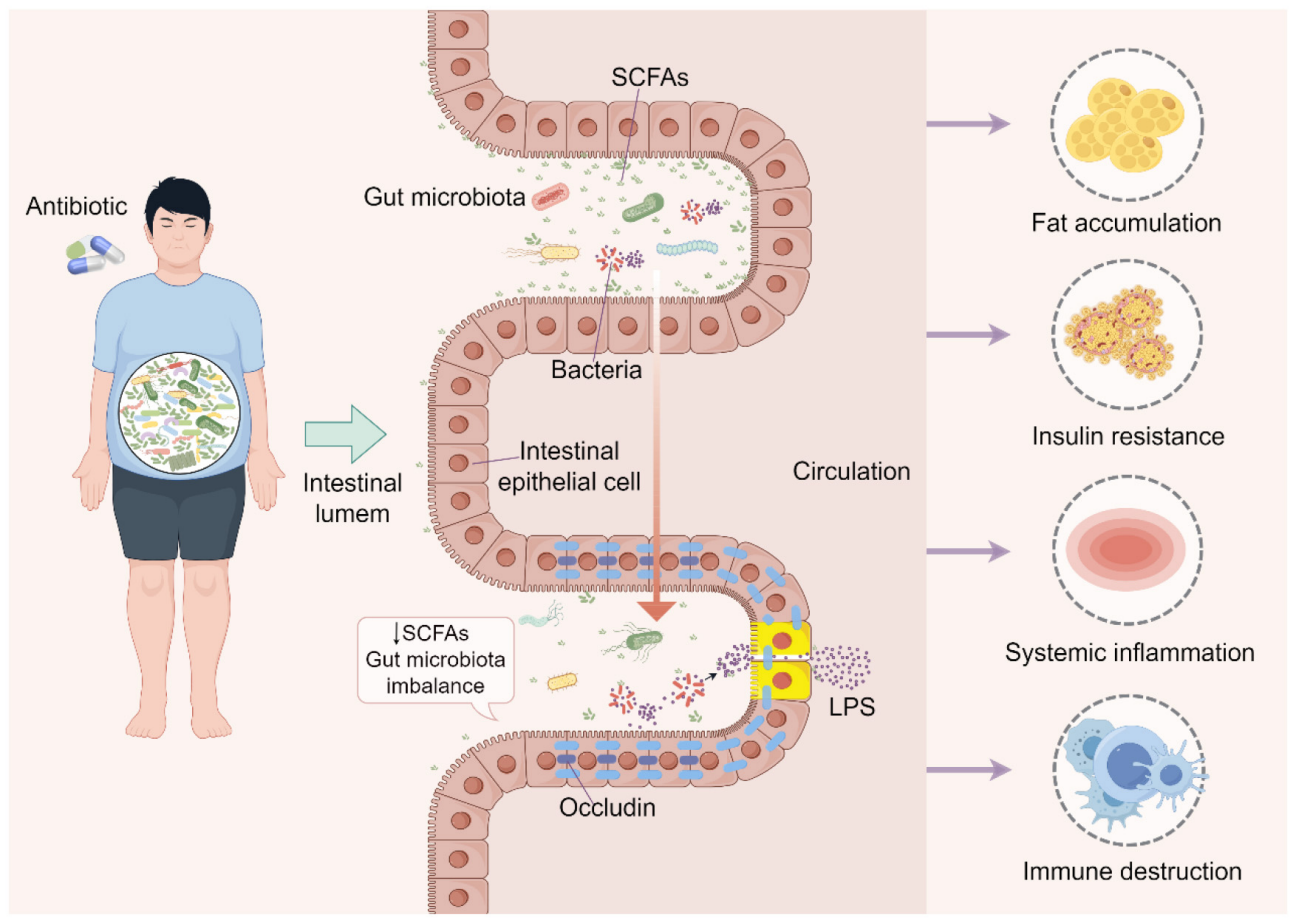
Antibiotics influence obesity and metabolic phenotypes through the gut microbiota.

Notably, antibiotic-induced disruption of the gut microbiota exhibits significant interindividual variability. In early life, overall microbial community changes following amoxicillin or amoxicillin-clavulanate exposure in healthy term infants are typically mild and transient (Benitez et al., 2025). In healthy volunteers, short-course intravenous administration of β-lactam antibiotics simultaneously disrupts bacterial communities, phage communities, and metabolic profiles, with abnormal microbial structures detectable even 90 days later. Concurrently, individuals with higher baseline β-lactamase activity demonstrated better metabolic recovery (d'Humières et al., 2024), suggesting that the gut's endogenous antibiotic degradation capacity may partially buffer the disruptive effects of antibiotics on the intestinal microbiota. In clinical populations, the effects of antibiotic exposure are often more complex, depending on antibiotic type, treatment duration, and individual baseline health status. Patients with chronic diseases frequently undergo long-term antibiotic therapy, and this prolonged exposure typically exerts a more pronounced effect on gut microbiota composition than short-term treatment in healthy volunteers (Rashidi et al., 2021). Recent studies have further demonstrated that antibiotics may influence host metabolic functions by disrupting gut microbiota composition. A metagenomic study of a preterm infant cohort revealed that short-term antibiotic exposure not only alters microbial composition but may also suppress or delay the production of key metabolites such as SCFAs (Xu et al., 2022). In animal models, although microbial composition partially recovered after short-term ceftriaxone treatment, differences in microbial interaction networks and metabolic pathways persisted during longer follow-up periods (Hong et al., 2024). These findings suggest that antibiotics may pose risks of long-term functional alterations by reshaping microbial interaction networks (Figure 1).

However, some reports indicate that antibiotic exposure has minimal or even no significant long-term effects on metabolism, a phenomenon that appears to be more pronounced in certain populations or under specific antibiotic usage scenarios (Benitez et al., 2025). These inconsistent findings may stem from several factors, including differences in study design, antibiotic type, dosage, exposure duration, and baseline microbiome composition. Such findings further highlight the complexity of the microbiome–host relationship while underscoring the need for more standardized controlled studies to elucidate the precise mechanisms and long-term outcomes of antibiotic exposure on metabolic health.

Furthermore, evidence suggests that severe depletion of the gut microbiota may, in certain circumstances, mitigate or even reverse adverse metabolic phenotypes. Suez et al. (Suez et al., 2014) found that antibiotic treatment eliminated glucose intolerance induced by artificial sweeteners, and this phenotype was transferable via microbiota transplantation, establishing a causal role for the microbiome in this process. Similarly, Suárez-Zamorano et al. (2015) confirmed that broad-spectrum antibiotic therapy improved glucose tolerance and insulin sensitivity while reducing white adipose tissue mass and promoting its browning. Notably, these metabolic improvements were reversed upon microbiota repopulation, further substantiating a microbiome-dependent mechanism. Collectively, these studies indicate that the metabolic consequences of antibiotic exposure exhibit strong context dependency, with effects potentially varying according to the degree of microbial depletion.

## 3 Antibiotic-induced metabolic cascade effects

### 3.1 Imbalance of energy metabolism and fat accumulation

Antibiotic exposure–related imbalances in energy metabolism and fat accumulation stem not merely from excessive energy intake, but from profound disruptions to the gut microbiota and host metabolic networks. The gut microbiota plays a crucial role in regulating host energy homeostasis. It influences host metabolism, fat storage, and appetite through several mechanisms that collectively contribute to maintaining energy balance. The microbiota produces metabolites such as SCFAs, bile acids, and amino acids, which act as signaling molecules to regulate energy metabolism, promote energy expenditure, and reduce fat accumulation (Zhang S. et al., 2024). Furthermore, the gut microbiota can influence host appetite and satiety signaling by modulating hunger-related hormones. Dysbiosis disrupts these hormonal signals, potentially leading to overeating and promoting obesity (Yu et al., 2024; Augustynowicz et al., 2025). In a mouse model, 3-week-old mice administered low-dose penicillin combined with a high-fat diet for 5 weeks exhibited higher body weight, abdominal fat, and blood glucose levels compared with mice fed a high-fat diet alone. A further study revealed that the antibiotic reduced phenyllactic acid (PLA), a metabolite derived from *Lactobacillus murinus*, thereby inhibiting the PPARγ signaling pathway in small intestinal epithelial cells. When researchers administered a high-fat diet combined with antibiotic intervention to mice with PPARγ specifically knocked out in small intestinal epithelial cells, no significant differences in body weight, abdominal fat, or blood glucose levels were observed compared with the high-fat-diet–only group (Shelton et al., 2023). These findings suggest that antibiotic exposure may increase obesity risk by reducing PLA production from *L. murinus* and inhibiting the PPARγ signaling pathway. Additionally, antibiotic exposure may influence adipose tissue thermogenesis through direct or indirect pathways, thereby reducing energy expenditure. An animal study revealed that the macrolide antibiotic azithromycin directly inhibits mitochondrial function and thermogenic gene expression (e.g., UCP1, PGC1α) in brown or beige adipocytes, thereby reducing cellular oxygen consumption. This effect correlates with decreased mitochondrial respiratory chain complex proteins and elevated reactive oxygen species levels (Yu et al., 2022).

Antibiotic exposure affects lipid metabolism through several mechanisms. SCFAs produced by the gut microbiota, such as butyrate and propionate, play a crucial role in regulating lipolysis, lipogenesis, and overall energy metabolism. These metabolites modulate adipocyte function and influence energy expenditure by activating G protein-coupled receptors (GPCRs), including GPR41 and GPR43 (May and den Hartigh, 2021). Furthermore, the Farnesoid X receptor (FXR) signaling pathway is closely linked to bile acid metabolism. FXR signaling regulates hepatic lipid metabolism by modulating gene expression associated with fatty acid synthesis and oxidation. Antibiotic exposure may disrupt the FXR signaling pathway, leading to increased fat accumulation in the liver and adipose tissue, thereby contributing to obesity and metabolic disorders (Meng et al., 2023). In juvenile mouse models, antibiotic-associated dysbiosis has been reported to potentially interfere with hepatic fatty acid oxidation through the FXR signaling pathway, a change that favors energy storage and fat accumulation (Wang et al., 2024). These signaling pathways form a complex regulatory network that collectively modulates energy balance. Antibiotic-induced alterations in the gut microbiota may disrupt this network, leading to energy dysregulation and increased fat accumulation.

### 3.2 Insulin resistance

Insulin resistance is a key phenotype in antibiotic-associated metabolic disorders and is typically driven by several mechanisms, including disruption of the intestinal barrier, systemic inflammation, and dysregulation of critical metabolic signaling pathways. Chronic low-grade inflammation is recognized as a major pathway contributing to insulin resistance. Antibiotic-associated alterations in microbial homeostasis may be accompanied by increased intestinal permeability, allowing bacterial products such as lipopolysaccharides (LPS) to translocate into the circulation. This process triggers metabolic endotoxemia and promotes the secretion of pro-inflammatory cytokines, including tumor necrosis factor-α (TNF-α) and interleukin-6 (IL-6). These inflammatory mediators interfere with insulin signaling and reduce insulin sensitivity through molecular mechanisms such as inducing serine phosphorylation of insulin receptor substrate 1 (IRS-1) (Zhang et al., 2025). Furthermore, antibiotic-induced reductions in SCFAs not only affect energy balance but also directly diminish insulin sensitivity. Butyrate, a key SCFA component, enhances intestinal barrier function, reduces endotoxin translocation, and directly improves peripheral insulin signaling (Zhang S. et al., 2024). A population-based study reported negative correlations between serum or fecal levels of butyrate and propionate and obesity-related and glycemic indicators (Zhang S. et al., 2024). Disruption of the bile acid–FXR signaling axis represents another critical pathway. Bile acids serve as key signaling molecules regulating glucose metabolism. When antibiotics alter gut microbiota composition, the deconjugation and conversion processes of bile acids are also disrupted. This alteration can influence the activation status of FXR and the G protein-coupled bile acid receptor TGR5 (Chen et al., 2024).

Furthermore, population-based multi-omics studies have revealed that individuals with insulin resistance often exhibit elevated levels of host-accessible monosaccharides in their feces, a phenomenon associated with enrichment of Lachnospiraceae bacteria in the gut. Excess monosaccharides produced by these bacteria may promote lipid accumulation and activate host inflammatory responses. Conversely, microbiota enriched in Bacteroidales effectively utilize these monosaccharides, exhibiting lower levels of inflammation and improved insulin sensitivity (Semo et al., 2024). These findings suggest that antibiotic-induced alterations in microbiota composition may impair the gut's ability to metabolize dietary carbohydrates, leading to accumulation of monosaccharides in the intestinal lumen and subsequent disruption of insulin signaling pathways.

### 3.3 Systemic inflammation and disruption of immune homeostasis

Antibiotic-induced disruption of local and systemic immune homeostasis in the gut is associated with compromised intestinal barrier integrity and diminished immunoregulatory signaling from the microbiota. Antibiotic exposure reduces the production of SCFAs, such as butyrate, which serves as the primary energy source for colonic epithelial cells and is critical for maintaining tight junctions between epithelial cells. Decreased butyrate levels weaken intestinal epithelial barrier function, leading to “leaky gut” (Takeuchi et al., 2023). Concurrently, antibiotic exposure may alter microbial composition and increase the relative abundance of potentially harmful bacteria. When compromised barrier function coincides with elevated levels of pro-inflammatory microbes, bacterial products such as LPS, flagellin, peptidoglycan, and even viable bacterial components can more readily translocate into the intestinal and portal venous circulation, thereby activating the immune system (Hu et al., 2020). These microbial antigens activate Toll-like receptors (TLRs) on immune cells (e.g., macrophages), thereby initiating inflammatory signaling pathways such as nuclear factor κB (NF-κB) and promoting the production of large amounts of pro-inflammatory cytokines (Liébana-García et al., 2025). This inflammatory response extends beyond the gut. Circulating LPS persistently stimulates immune cells in peripheral metabolic organs such as the liver and adipose tissue, establishing a chronic low-grade inflammatory state that is directly implicated in insulin resistance and adipose tissue dysfunction (Han et al., 2025). Ni et al. (2025) observed reduced expression of multiple immune-related genes in the ileum of tylosin-exposed mouse models, concurrent with activation of pro-inflammatory lipid metabolism pathways in the liver, supporting a potential link between intestinal immune suppression and systemic inflammatory metabolic dysfunction.

Furthermore, a study indicates that antibiotic-associated dysbiosis may be accompanied by reduced numbers or impaired function of colonic regulatory T cells (Tregs) (Zhang et al., 2021). Tregs are crucial for maintaining immune tolerance and suppressing excessive inflammatory responses. Their depletion further diminishes immune tolerance, leading to immune dysregulation. This immune imbalance can exacerbate systemic inflammatory responses while simultaneously increasing the risk of obesity, diabetes, and other metabolic disorders. Cox et al. (2014) found that low-dose penicillin exposure during infancy suppressed the expression of multiple immune-related genes in the ileum, including pathways involved in Th17-related responses and antimicrobial defense. These findings suggest that antibiotic-induced microbiome disruption during infancy may impair intestinal immune maturation and affect immune homeostasis.

### 3.4 What multi-omics has revealed about antibiotic–microbiome–metabolism links in obesity

In recent years, advances in metagenomics have deepened our understanding of how antibiotic exposure alters the gut microbiota at the genetic level. Antibiotics can induce changes in the microbial gene pool, leading to alterations in microbial metabolism and function. They promote the expansion of resistant microbial populations, through which antimicrobial resistance genes (ARGs) can spread between differenst microorganisms. This process alters the microbiota's ability to metabolize nutrients, thereby affecting the host's metabolic state (Zhang Y. et al., 2024; Li et al., 2023). A study revealed that even short-term ciprofloxacin use in healthy adults can confer resistance to certain commensal bacteria, with this resistance persisting for weeks after discontinuation (Yaffe et al., 2025). This finding indicates that the impact of antibiotics extends beyond transient microbial abundance shifts and may drive adaptive evolution within the microbiota. At the population level, per capita antibiotic consumption in different countries correlates closely with the abundance of mobile drug resistance genes in the gut (Lee et al., 2023), suggesting that widespread antibiotic use may alter the structure of gut resistance genes at the population level. Studies based on long-term prescription data have further revealed significant differences in the duration of effects among different classes of antibiotics on the gut microbiota. For example, changes induced by macrolides and lincosamides can persist for years, whereas the effects of certain β-lactams are relatively transient (Mulder et al., 2020). Notably, clinical trials indicate that even after discontinuation, although overall microbial composition tends to recover, resistance genes may persist at elevated levels for extended periods (Dhariwal et al., 2023), demonstrating a dissociation between restored microbial structure and persistent resistance traits.

Metabolomics studies further reveal that antibiotic-induced microbial alterations affect the production of key metabolites such as SCFAs, bile acids, and amino acids (Zhang S. et al., 2024). These metabolites play crucial roles in regulating lipid metabolism, insulin sensitivity, and inflammatory responses. SCFAs are essential for maintaining intestinal barrier integrity and regulating energy metabolism; their depletion may weaken their protective effects on host health (Takeuchi et al., 2023). Notably, reduced butyrate levels are frequently associated with heightened inflammation and impaired adipocyte function, changes that may exacerbate obesity and insulin resistance.

Integrated analyses of metagenomics and metabolomics provide a more comprehensive perspective for understanding the effects of antibiotics on host metabolism. Antibiotic-induced microbial changes and subsequent microbial evolutionary processes may determine whether the gut microbiota gradually recovers to its original state or establishes a new, relatively stable configuration, thereby providing a sustained microbial context for subsequent metabolic cascade effects (de la Cuesta-Zuluaga et al., 2024).

## 4 Early-life antibiotic exposure

Early-life antibiotic exposure, particularly during infancy, has attracted considerable attention because this period represents a critical stage of gut microbiota development. Antibiotic exposure during this pivotal window may disrupt the establishment of the gut microbiota and is associated with increased risks of subsequent obesity, immune dysfunction, and metabolic disorders.

A prospective cohort study conducted in New Zealand involving 5,128 children analyzed the relationship between early-life antibiotic exposure and weight status at age 4.5 years. The results indicated that recurrent antibiotic exposure was associated with higher BMI and an increased risk of obesity, with this association being particularly pronounced when antibiotic exposure occurred within the first 12 months of life (Grant et al., 2020). Further studies have revealed that antibiotic exposure can significantly affect the structure and function of the infant gut microbiota. Observational evidence indicates that early-life antibiotic exposure is associated with reduced levels of beneficial gut bacteria, including *Bifidobacterium* and *Lactobacillus*. Concurrently, it may promote the proliferation of potentially pathogenic bacteria, such as an increased relative abundance of *Enterobacteriaceae*. High-throughput sequencing analyses have further shown that antibiotic exposure correlates with reduced gut microbiota diversity and altered microbial composition, particularly in preterm infants receiving antibiotics. In these infants, the abundance of the phylum *Proteobacteria* increases, while the relative abundance of the phylum *Firmicutes* decreases (Gong et al., 2021).

Early-life antibiotic exposure may exert long-term effects on infant metabolic health by altering gut microbiota composition, promoting lipid metabolism disorders, and increasing obesity risk. Mouse models investigating the combined effects of high-fat diets and low-dose penicillin exposure demonstrated that antibiotic exposure synergistically amplified weight gain and fat accumulation in the presence of a high-fat diet, with these effects persisting even after antibiotic cessation (Nieuwdorp and Rios-Morales, 2023). Early antibiotic exposure may further influence metabolic health by altering immune system function. Lamont et al. (2020) examined the relationship between antibiotic use during pregnancy, delivery, and the neonatal period and the development of childhood inflammatory and metabolic diseases. Their findings indicated significant associations between antibiotic exposure and childhood allergic diseases, asthma, and obesity, with the strongest associations observed between prenatal antibiotic exposure and the risk of childhood obesity and allergic diseases.

Furthermore, a randomized controlled trial involving 147 infants born at ≥36 weeks' gestation found that antibiotic exposure promotes the accumulation of antibiotic resistance genes through selective pressure. This accumulation not only threatens gut microbiota health but may also pose long-term antimicrobial resistance risks (Reyman et al., 2022). A cohort study also revealed that cesarean-delivered infants typically carry higher antibiotic resistance gene loads, particularly within the first 6 months of life (Jokela et al., 2024). These findings suggest that early-life antibiotic exposure may increase the reservoir of resistance genes by altering gut microbiota composition. Such effects are not merely transient; they may exert long-term impacts on infant health by disrupting gut microbiota stability and facilitating the transmission of resistance genes.

## 5 Probiotic-mediated microecological restoration strategies

Probiotics, as an important means of microecological regulation, hold significant value in restoring antibiotic-associated dysbiosis and alleviating obesity and metabolic abnormalities. Fecal microbiota transplantation (FMT) is considered a comprehensive approach for restoring gut microbiota diversity. However, its clinical application remains constrained by several factors, including safety concerns, donor variability, regulatory barriers, and the potential risk of transmitting pathogenic microorganisms (DeFilipp et al., 2019; Zmora et al., 2018). In contrast, probiotic formulations consist of specific strains with well-defined safety profiles and standardized production, making them more suitable for broader clinical applications, particularly in non-life-threatening conditions such as metabolic disorders.

Probiotics can restore intestinal health by regulating the composition of the gut microbiota, promoting the growth of beneficial bacteria such as *Lactobacillus* and *Bifidobacterium*, and inhibiting the proliferation of harmful bacteria such as *Enterococcus* (Klancic and Reimer, 2020). Studies indicate that probiotic supplementation can increase the production of SCFAs, metabolites produced by the gut microbiota, reduce antibiotic-induced damage to the intestinal barrier, thereby decreasing fat accumulation and improving lipid metabolism (Vallianou et al., 2020). In mouse models, the probiotic strains *Lactobacillus plantarum* L11 and *Lactobacillus reuteri* LR were found to reduce fat accumulation and partially improve obesity-related metabolic abnormalities by regulating gut microbiota and enhancing lipid metabolism. Specifically, *L. plantarum* L11 and *L. reuteri* LR mitigate obesity and fat accumulation by activating the AMPK signaling pathway, promoting fatty acid oxidation, and enhancing lipolysis (Liang et al., 2024). In animal models and some clinical studies, probiotic supplementation has been shown to improve insulin sensitivity, reduce visceral fat accumulation, and regulate appetite-related hormones such as glucagon-like peptide-1 (GLP-1) and peptide YY (PYY) (Green et al., 2020; Feng et al., 2020). The combined application of probiotics and prebiotics (synbiotics) not only modulates gut microbiota composition but also enhances their therapeutic effects against obesity by promoting adipose tissue browning, thereby increasing energy expenditure (Sergeev et al., 2020).

However, the efficacy of probiotics exhibits strong strain specificity, and their clinical benefits are not universally consistent. Multiple studies have specifically evaluated the effects of probiotic supplementation following antibiotic treatment. Results indicate that probiotics can partially restore microbial diversity while alleviating antibiotic-associated diarrhea and modulating inflammatory markers. However, recovery of the original microbiota is often incomplete, and probiotic strains typically struggle to achieve long-term colonization in the host gut (Suez et al., 2018; Hempel et al., 2012). In the field of obesity treatment, meta-analyses indicate that probiotic supplements may yield modest reductions in body weight and BMI, but their overall effect size remains relatively small compared with dietary or pharmacological interventions (Borgeraas et al., 2018). Some clinical studies suggest that probiotics exert certain beneficial effects on obesity and metabolic health; however, other reports document limited efficacy or even no discernible effect (Cao et al., 2024; Hasani-Ranjbar et al., 2024). This inconsistency may be attributed to several factors, including strain type, dosage, intervention duration, and individual baseline microbiome status (Pontes et al., 2025; Caesar, 2025). Certain strains may be more suitable for specific antibiotic-induced dysbiosis, whereas others may have minimal or insignificant effects on metabolic health (Torres et al., 2024). Furthermore, the regulatory framework surrounding probiotics remains complex, and standardized guidelines for their clinical use are still limited.

In summary, probiotics, as an important microecological intervention strategy, may alleviate antibiotic-associated dysbiosis and related metabolic disorders through multiple pathways, including regulating gut microbiota, improving immune responses, and promoting lipid metabolism (Fuochi and Furneri, 2023), thereby demonstrating potential in obesity prevention and treatment. However, their clinical application still faces several challenges. Factors such as individual response variability, strain selection, dosage, and intervention duration may all influence probiotic efficacy (Montassier et al., 2021). Therefore, future research should further explore strategies to optimize probiotic use, particularly in specific populations.

## 6 Conclusion

Antibiotic exposure can trigger a series of metabolic cascade effects by disrupting the structure and function of the gut microbiota, thereby influencing host energy metabolism, lipid deposition, and immune homeostasis. Evidence suggests an association between early-life antibiotic exposure and increased risk of childhood obesity, while animal studies further support the causal role of the gut microbiota in this process. However, the metabolic effects of antibiotics exhibit substantial context dependency, with outcomes influenced by multiple factors, including antibiotic class, dosage, exposure window, host developmental stage, and baseline microbiota composition.

Although recent advances in multi-omics technologies have provided new perspectives for elucidating the complex interactions among antibiotics, the microbiome, and host metabolism, several key scientific questions remain unresolved. First, most existing population studies are observational, making it difficult to fully exclude potential confounding factors such as infection severity, dietary habits, and socioeconomic status. Therefore, more rigorously designed prospective cohort studies and well-controlled intervention studies are needed to clarify causal relationships. Second, the long-term differential effects of various antibiotic classes on the gut microbiota and host metabolism require systematic evaluation, particularly during critical developmental stages in early life. Furthermore, current evidence regarding microbiome-based interventions (such as probiotics, synbiotics, and fecal microbiota transplantation) for antibiotic-associated metabolic abnormalities remains limited. Optimal strain combinations, timing of intervention, and target populations require further investigation.

Future research should integrate multi-omics approaches—including metagenomics, metabolomics, and immunomics—to systematically elucidate the causal links between microbial functional remodeling following antibiotic exposure and host metabolic alterations. Meanwhile, promoting rational antibiotic use in clinical practice and developing personalized microbiome-based intervention strategies may provide novel avenues for preventing and treating obesity and related metabolic disorders.
